# Collagen Type XI Alpha 1 (COL11A1): A Novel Biomarker and a Key Player in Cancer

**DOI:** 10.3390/cancers13050935

**Published:** 2021-02-24

**Authors:** Sameera Nallanthighal, James Patrick Heiserman, Dong-Joo Cheon

**Affiliations:** Department of Regenerative and Cancer Cell Biology, Albany Medical College, Albany, NY 12208, USA; nallans@amc.edu (S.N.); heiserj@amc.edu (J.P.H.)

**Keywords:** collagen, COL11A1, biomarker, cancer-associated fibroblasts, metastasis, chemoresistance

## Abstract

**Simple Summary:**

Collagen type XI alpha 1 (COL11A1) is a part of type XI collagen, which is important for bone development. Interestingly, COL11A1 levels are frequently upregulated in various cancers and high levels of COL11A1 are correlated with poor clinical outcome in many solid cancers. Increasing evidence shows that COL11A1 promotes tumor cell aggressiveness through multiple mechanisms. In this review, we discuss how COL11A1 serves as both a biomarker and a key player in cancer. This knowledge will facilitate the development of novel therapies to treat COL11A1-high cancers.

**Abstract:**

Collagen type XI alpha 1 (COL11A1), one of the three alpha chains of type XI collagen, is crucial for bone development and collagen fiber assembly. Interestingly, COL11A1 expression is increased in several cancers and high levels of COL11A1 are often associated with poor survival, chemoresistance, and recurrence. This review will discuss the recent discoveries in the biological functions of COL11A1 in cancer. COL11A1 is predominantly expressed and secreted by a subset of cancer-associated fibroblasts, modulating tumor-stroma interaction and mechanical properties of extracellular matrix. COL11A1 also promotes cancer cell migration, metastasis, and therapy resistance by activating pro-survival pathways and modulating tumor metabolic phenotype. Several inhibitors that are currently being tested in clinical trials for cancer or used in clinic for other diseases, can be potentially used to target COL11A1 signaling. Collectively, this review underscores the role of COL11A1 as a promising biomarker and a key player in cancer.

## 1. Introduction

Collagens are the most abundant proteins (~30% mass) in mammals and the main component of extracellular matrix (ECM) [1]. Collagens comprise 28 subtypes (type I through XXVIII) and type I collagen is the most abundant type (~90%) in the body [1]. Each collagen can form a homotrimer or heterotrimer consisting of three alpha chains. Each alpha chain is synthesized as a procollagen containing N-terminal and C-terminal propeptides and forms a triple helix in the cytoplasm. Once secreted, both N- and C-terminal propeptides are cleaved by proteinases, crosslinked, and assembled into collagen fibrils [2,3].

COL11A1 encodes one of three alpha chains of type XI collagen, a minor fibrillar collagen mainly expressed in the cartilage [3,4]. In the cartilage, COL11A1 forms a heterotrimer with COL11A2 and COL2A1 to assemble type XI collagen [3,4]. Mutations in COL11A1 gene are associated with type II Stickler syndrome and Marshall syndrome, two autosomal dominant disorders showing varying degrees of facial dysmorphism, nearsightedness, and hearing loss [5,6,7,8]. A single-nucleotide polymorphism in COL11A1 gene is also associated with susceptibility to lumbar disc herniation [9]. The homozygous chondrodysplasia (cho/cho) mice harboring a point mutation in COL11A1 gene die at birth due to severe skeletal defects [10]. Furthermore, collagens in the cartilage of cho/cho mice form abnormally thick and fragmented fibers [11,12], demonstrating crucial roles of COL11A1 in nucleation and initial assembly of collagen fibers.

**Figure 1:**
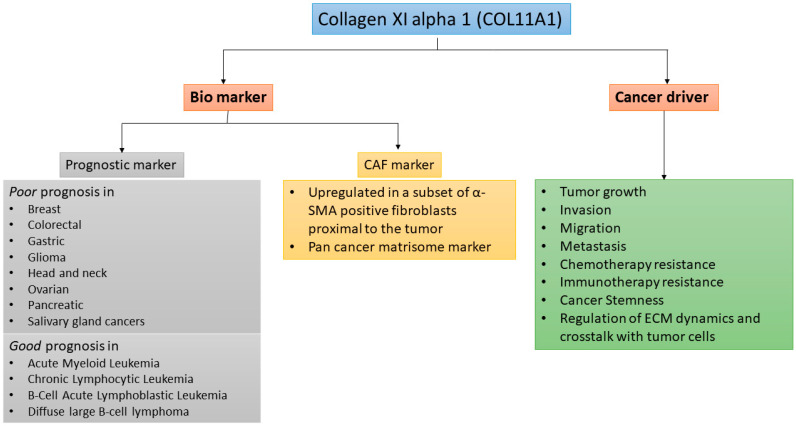
COL11A1 is a biomarker and is driver of aggressiveness in cancer.

Although COL11A1 expression in normal tissues is very low, COL11A1 expression is significantly upregulated in many types of cancer [2,13] (Figure 1). High levels of COL11A1 are often associated with aggressive tumor phenotype and poor prognosis in multiple solid tumors types such as ovarian, breast, pancreas, and colorectal cancer [2,13]. In stark contrast, it has been shown in hematological malignancies including Acute Myeloid Leukemia (AML), Chronic Lymphocytic Leukemia (CLL), B-Cell Acute Lymphoblastic Leukemia (B-ALL), and Diffuse Large B-cell Lymphoma (DLBCL) that COL11A1 overexpression is associated with better prognosis [14]. In solid tumors, although a small number of cancer cells overexpress COL11A1, COL11A1 is predominantly overexpressed by a subset of cancer-associated fibroblasts (CAFs) adjacent to cancer cells [2], suggesting COL11A1 as a specific marker for CAFs. However, despite the importance of COL11A1 in skeletal development and fibrillogenesis, its biological functions in cancer remain poorly understood.

In this review, we will provide a comprehensive overview of the biological functions of COL11A1 in cancer and discuss how COL11A1 mediates the crosstalk between cancer cells and the tumor microenvironment (TME) to regulate cancer cell phenotype. We will also discuss how COL11A1 can serve as a promising biomarker and therapeutic target to treat cancer as well as the remaining challenges to address our knowledge gaps in COL11A1 biology.

## 2. COL11A1 Biology and Binding Partners

COL11A1 binds to COL11A2 and COL11A3 to form a heterotrimeric complex of collagen type XI [15]. More recent studies on type XI collagen show that the COL11A3 subunit is actually the product of the COL2A1 gene [16]. It is now accepted that collagen type XI is a triple helical heterotrimer made up of COL11A1, COL11A2, and COL2A1. It should be noted that to date, no study has confirmed the existence of a triple helical homotrimer version of type XI collagen, and the composition of collagen type XI might change in a tissue specific manner. Collagen type XI has been known to regulate collagen type II fibrillogenesis across different mammalian model organisms [3,12,17] and is usually associated with thin collagen type II fibers in cartilage. Collagen type XI has also been shown to regulate collagen type I fibrillogenesis in chick embryo sternal chondrocytes as well [18]. However, COL11A2 and COL2A1 are not the only collagen subunits that can bind to COL11A1. A study by Yoshioka et al. [19] shows high expression of COL11A1 in tongue, the intestine, and the optic vesicle of the developing mouse embryo, in which COL5A2 is also highly expressed. COL11A1 is expressed in equal ratios to COL5A1 and COL5A2 in bovine bone as demonstrated by Niyibizi and Eyre [20]. The authors of this study suggest that COL11A1 can form a collagen heterotrimer with COL5A1 and COL5A2 while also noting the possibility that other subunit combinations can be possible (such as one COL11A1 and two COL5A1 subunits, for example). In agreement with these studies, Kleman et al. [21] demonstrate that COL11A1 may bind to COL5A2 in a cancer context as well. This groups show that A204 cells (a human rhabdomyosarcoma cell line) deposit a collagen matrix made up of COL11A1 and COL5A2 at a 2:1 ratio, suggesting that these alpha chains form a heterotrimer at those ratios. Although the authors hypothesize that COL11A1 forms a heterotrimer with COL5A2 at a 2:1, other combinations of these collagen alpha chains might be possible in a cancer type-specific manner.

COL11A1 may also bind to many other proteins outside of collagens. Using mass spectrometry, Brown et al. [22] identified several proteins that interact with the amino terminal domain (NTD) of COL11A1 in fetal bovine cartilage, which is displayed at the surface of collagen fibrils in cartilage. These proteins include ECM proteoglycans (such as biglycan, fibromodulin, chondroadherin) and other ECM components (Thrombospondin-1, matrilin-1/3, chondrocalcin) in addition to other collagens (collagens type II, XI, XIV, XII, and IX). Some cellular proteins also associate with COL11A1’s NTD, including cell surface proteins (Annexins I, II, V) and protein fate and modification proteins (Heat shock proteins HSPA5 and HSP90B1), and metabolic and cytoskeletal proteins (Fructose bisphosphate aldolase, lactate dehydrogenase A, β-actin). Oncostatin M (OSM), an inflammatory cytokine, has also been shown to bind to collagen type XI after being deposited by neutrophils to MDA-MB-231 breast cancer cell-derived matrices in vitro [23]. Although this interaction is not known to regulate COL11A1 function, binding, or signaling, OSM immobilized to type XI collagen induced Signal Transducer and Activator of Transcription (STAT) signaling in the breast cancer cell line T47D.

Taken together, COL11A1 can bind to COL11A2 and COL2A1 to form collagen type XI, which is known to regulate fibrillogenesis of collagen types I and II. There is also evidence that COL11A1 can also bind to COL5A1 and COL5A2 to form its own unique heterotrimer, although it is unclear at what ratios COL11A1 may associate with these two collagen V subunits. In addition to other collagens, COL11A1 may also bind to other proteins, such as proteoglycans, other components of the ECM, and potentially even some cellular proteins, including the cytokine OSM.

Overall, it is unclear how the composition of type XI collagen varies in the context of cancer compared to that in normal tissues. Given that the TME undergoes extensive reorganization and alteration in collagen composition, it is possible that COL11A1 can partner with unconventional binding partners to promote malignancy, which, at this point, remains unexplored.

## 3. COL11A1 in Tumor Microenvironment

### 3.1. CAFs Are the Major Source of COL11A1

CAF activation is associated with the acquisition of a partial myofibroblast-like phenotype often seen in diseases such as cancer and chronic inflammation. This is associated with an increase in ECM deposition, fibrosis, and cytokine secretion. COL11A1 overexpression has only been observed in desmoplastic areas of the tumors composed majorly of CAFs in different cancers [14,24,25,26,27] but not in fibroblasts in inflammatory diseases, making COL11A1 a unique marker for CAFs. In fact, Kleinert et al. show that COL11A1 expression is a very accurate biomarker that discriminates pancreatic cancer from pancreatic chronic inflammation [28]. Immunohistochemical studies in pancreatic and ovarian cancers identify a subset of CAFs marked by high COL11A1 expression [2,13,14,24,25]. In particular, in aggressive ovarian cancer, COL11A1 expression is limited to the αSMA-positive CAFs adjacent to the tumor periphery (<1 mm from the epithelial cells) or within the tumor, suggesting that COL11A1 expression can be stimulated in CAFs when they are in close proximity to cancer cells [14,24]. Furthermore, it has been shown that normal fibroblasts can be “educated” to express COL11A1 by co-culturing them with cancer cell lines, further solidifying the idea that the interplay between cancer cells and fibroblasts is essential for malignant progression [14]. COL11A1 expression in CAFs is associated with co-expression of a panel of EMT- and stemness-regulating genes across 13 different cancer types. Interestingly, Ingenuity pathway analysis of these COL11A1-correlated genes placed TGFβ1 as a master regulator of the pan-cancer COL11A1-correlated genes and COL11A1-associated biological functions [14]. This is in line with other studies that demonstrate the role of TGFβ1 signaling in driving COL11A1 expression, EMT phenotype, and cancer invasion and metastasis [14,24,25,27,29,30,31,32,33,34]. Overall, these studies show that CAFs are the major source of COL11A1 in tumors.

### 3.2. COL11A1 Expression in Other Cancer-Associated Cells

Cancer is a highly plastic disease where different cells in the TME alter their identity to aid in cancer growth in response to various external stimuli. Given the role of COL11A1 in mediating crosstalk between different cell types in the TME, it is likely that COL11A1 might play a role in promoting plasticity in different cell types in the TME. In line with this hypothesis, it was identified that CAFs exhibiting the “metastasis signature” composed of COL11A1, INHBA (inhibin beta A), THBS2 (thrombospondin 2), originate from adipocytes when the cancer metastasizes to a fat-rich microenvironment [31,35]. The authors performed extensive computational analyses of single-cell RNAseq data across many cancer types, to identify that naturally occurring normal human adipose-derived stromal cells transform into COL11A1-expressing CAFs [35]. This data suggests that the interaction between tumor cells and the microenvironment drives the transformation of different stromal cells into COL11A1-expressing CAFs to promote malignancy. Similarly, Galvan et al. show that other cell types such as human bone marrow-derived mesenchymal cells express COL11A1 [29]. It has been reported that human bone marrow-derived mesenchymal cells can differentiate in vitro into cancer-promoting fibroblast/myofibroblast-like cells when (i) cocultured with/or in the conditioned medium from cancer cells (ii) stimulated with TGF-β1 [36,37,38]. In this study, the authors show that COL11A1-expressing colon CAFs resemble a myofibroblast-like phenotype, similar to the COL11A1-overexpressing phenotype observed in TGF-β1-stimulated human bone marrow-derived mesenchymal cells. These findings support the hypothesis that some of the COL11A1-expressing CAFs originate from COL11A1-expressing human bone marrow-derived mesenchymal cells [29]. In another study, Buckanovich et.al. show that COL11A1 expression can be elevated in intratumoral vasculature in ovarian cancer [39]. When compared to respective normal tissues, the authors demonstrate elevated levels of COL11A1 expression in tumor endothelial cells, which is associated with poor outcomes in different cancer types [39]. Although not explored in this study, it is an intriguing possibility that COL11A1-expressing bone marrow-derived mesenchymal cells differentiate into COL11A1-expressing tumor endothelial cells upon contact with cancer cells. The phenomenon of bone marrow-derived mesenchymal cells differentiating into tumor endothelial cells has been previously reported [40].

Fibrosis resulting from excessive ECM deposition is induced by activated stellate cells and is an integral part of pancreatic diseases like chronic inflammation and cancer. It has been postulated that stellate cells may be derived from mesenchymal stem cells, or tissue resident fibroblasts in a cancer setting. Genome-wide transcriptional analysis in human pancreatic ductal adenocarcinoma identified that activated pancreatic stellate cells are yet one more type of cancer-associated cells that overexpress COL11A1 [41]. However, this study does not further elucidate the functions of COL11A1 in stellate cells or whether COL11A1 is required for the activation of stellate cells. Nevertheless, activated pancreatic stellate cells have been shown to mediate poor prognosis, chemoresistance, immunosuppression, and metabolic reprogramming in cancer [42].

Overall, human mesenchymal cells, tumor endothelial cells, and pancreatic stellate cells are the other cell types that show elevated COL11A1 expression. Given that COL11A1 expression is specific to cancer-associated stromal cells, targeting COL11A1 will significantly enhance the therapeutic specificity and reduce the risk of off-target effects. 

### 3.3. COL11A1 Expression in the Matrisome Is a Pan-Cancer Biomarker

The ECM proteome, or “matrisome,” of cancers has been an area of extensive research recently. Matrisome is defined as a collection of genes encoding core ECM proteins and ECM-associated proteins. The core matrisome is composed of about 300 genes encoding ECM glycoproteins (laminins, tenascins, thrombospondins, fibrillins, fibronectin, collagens, proteoglycans, etc.), and about 700 matrisome-associated genes [32,34]. Multiple studies that analyzed human pan-cancer matrisome demonstrate that COL11A1 is one of the top genes upregulated in late stage aggressive cancers. For example, Yuzhalin et al. devised a 9-core-matrisome-gene signature (COL11A1, COL10A1, COL1A1, AGRN, BGN, COMP, MFAP2, MXRA5, SPP1) consistently up-regulated in breast, esophageal, gastric, lung, ovarian, and colorectal adenocarcinoma tissue sections [34]. Another study by Lim et al. (2017) in non-small cell lung cancer (NSCLC) also identified a 29-gene classifier of tumor matrisome (here on out referred to as COL11A1-matrisome signature) that predicts poor clinical outcome and drug resistance in patients where COL11A1 is the top upregulated protein [43,44]. Given these findings, it highly possible that the patterns of deregulated matrisome genes are conserved in various cancer types. Furthermore, in breast, colorectal, and pancreatic cancer, there is a progressive increase in the expression of the COL11A1-matrisome signature during tumor development from normal to adenoma to carcinoma in-situ, and further to invasive carcinoma [44]. Another pan-cancer analysis study elegantly used multi-omics and bioinformatics analysis to identify core matrisome signature and its association with clinical outcome. Not surprisingly, COL11A1 is one of the four core matrisome proteins upregulated across all cancer types and correlates positively with worst clinical outcome in all cancer types tested [45]. Taken together, these findings highlight the role of COL11A1 as a pivotal molecule that regulates cancer aggressiveness and the possible commonality in the deregulation of tissue architecture that drives cancer progression across multiple cancer types.

It is interesting to note that across multiple cancer subtypes, Lim et.al identified that the COL11A1-matrisome signature is upregulated in both epithelial and stromal CAFs, once again suggesting the crosstalk between CAFs and tumor epithelial cells to instruct one another to shape tumor-promoting matrisome [43]. CAF targeting so far had limited success owing to lack of specific markers and systemic toxicity. Therefore, the COL11A1-matrisome genes may provide specific targets that are of relevance for this purpose.

### 3.4. Mechanical Properties of COL11A1-Rich ECM

It is well documented that an increase in tissue stiffness can modify mechanotransduction resulting in the reorganization of the ECM. Reorganization of ECM by cancer cells is a critical phenomenon that regulates several key cascades including tumor progression, drug resistance and metastasis. To investigate whether the presence of certain matrisome proteins can contribute to dynamic changes in mechanical and biochemical properties of tumor matrix, Pearce et al. [45] measured gene expression, matrisome proteomics, cytokine and chemokine levels, cellularity, ECM organization, and biomechanical properties in matched minimal and extensive high-grade serous ovarian cancer. They show that among several factors that drive malignancy, COL11A1 is the only collagen gene upregulated in the matrisome of aggressive cancer. There is also a positive correlation between the stage of cancer and COL11A1 expression, COL11A1 expression and matrix stiffness, disease progression and increased ECM [45]. Furthermore, using IHC, it has been confirmed that COL11A1 is also upregulated in conjunction with other ECM proteins that promote collagen organization and ECM stiffness (fibronectin, cathepsin B, and cartilage oligomeric matrix protein), in three tissue microarrays from triple-negative breast cancer (TNBC), pancreatic ductal adenocarcinoma (PDAC), and diffuse large B-cell lymphoma [45]. Linear collagen fibrils have been previously shown to provide tracks for migration of cancer cells and might aid in metastasis [46]. In line with this study, it has been shown that in the ECM of ovarian cancer, COL11A1 is aligned in thin linear collagen fibrils whereas type I collagen forms dense wavy collagen fibrils [14]. This suggests that COL11A1-rich ECM might increase migration of cancer cells. Taken together, it is very likely that COL11A1 alters the mechanical properties of the ECM to increase tumor aggressiveness.

## 4. COL11A1 in Tumor Cell Migration and Metastasis

COL11A1 expression is absent in benign pathological conditions involving desmoplasia such as hyperplasia, fibrosis, cirrhosis, pancreatitis and inflammatory bowel disease or premalignant lesions [2,13]. Interestingly, in invasive carcinomas, high expression of COL11A1 is detected in the stroma of breast, colorectal, esophagus, glioma, gastric, lung, ovarian, pancreatic and salivary gland cancers [14,24,25,26,27,29,33,47,48,49,50,51,52] (Table 1, Figure 1). Moreover, in these cancers, the expression of COL11A1 positively correlates with progression and lymph node metastasis. A multi-cancer gene expression analysis performed to identify a transcriptional signature commonly expressed by most cancer cells when they switch to an invasive phenotype termed “metastasis signature”, also underscore COL11A1 as one of the proteins potentially regulating invasion and metastasis (gene list: COL11A1, INHBA, THBS2) [31]. A pan-cancer gene expression study shows that several genes of COL11A1-correlated gene set are drivers of the metastatic program, EMT, and are co-expressed in COL11A1-expressing CAFs [14]. While the majority of these studies focused on stromal COL11A1, a few studies have shown that aggressive tumor cells driving metastasis and drug resistance also express high levels of COL11A1. Using patient derived-tumor sections and adjacent normal tissues, Zhang et al. demonstrate that esophageal cancer cells express high levels of COL11A1, which correlate with advanced tumor grade and lymph node metastasis [51]. Xenograft mouse models and cell lines derived from lung metastasis in renal carcinoma [53], breast cancer tissues [54], lung cancer tissues and cell lines [47,50], and drug-resistant and invasive cell lines and xenografts of ovarian cancer [33] also show elevated COL11A1 expression. Whether COL11A1 originated from stromal cells activates different signaling compared to tumor cell intrinsic COL11A1 is still unclear. However, regardless of the origin, there is undisputed evidence to link tumor or stromal COL11A1 to cancer invasiveness and metastasis.

Although there are functional studies confirming the role of COL11A1 as a contributor of metastasis and invasion, there is a limited understanding of the mechanisms underlying COL11A1-mediated metastasis. A few studies listed here provide a brief understanding of the signaling pathways that drive COL11A1-mediated metastasis. In ovarian, gastric, esophageal, pancreatic, lung, and HNSCC cell lines si/shRNA-mediated knockdown of COL11A1 significantly abrogates the invasive potential of cancer cells [33,47,49,50,51] (Table 1). Furthermore, COL11A1-knockdown human ovarian cancer cell lines are defective in inducing peritoneal metastasis and lung colonization when inoculated into mice [33]. Matrix metalloproteinase 3 (MMP3) has been identified as one of the targets of COL11A1 signaling in this study [33]. Another study in a mouse model of pancreatic cancer suggests that COL11A1 can be activated by the Hedgehog signaling pathway [68]. In xenograft mouse models of breast cancer, COL11A1 expression is negatively regulated by transcription factor CDX2 and microRNA let-7b and the loss of COL11A1 expression by upregulating CDX2 let-7b suppressed metastasis [55]. Muscle, intestine and stomach expression 1 (Mist1), a transcriptional repressor can inhibit COL11A1 expression and reverse EMT in a pancreatic cancer mouse model [67]. It should be noted that majority of these mechanistic studies were performed in COL11A1-overexpressing cancer cells which only contribute to a minor part of COL11A1 signal in the tumor setting. There is no evidence whether CAFs, the major source of COL11A1 also activate similar signaling pathways to drive COL11A1 signaling. Growth factor signaling mediates crosstalk between cancer cells and CAFs. For example, secretion of TGF-β1 from cancer cells can transform stromal fibroblasts into CAFs. CAFs, in turn, can secrete high amounts of TGF-β1 which further stimulate tumorigenesis, migration, invasion and metastasis. Indeed, TGF-β1 signaling pathway can also regulate COL11A1 expression through transcription factor NF-Y in human ovarian cancer cell lines in vitro and in vivo [33]. Wu et al. show that blocking TGF-β1 drastically reduces not only COL11A1 expression but also COL11A1-dependent invasion in human ovarian cancer cell lines. The role of TGF-β1 and other potential regulators of COL11A1 will be discussed in detail in the later sections.

## 5. COL11A1 in Cancer Drug Resistance

### 5.1. Role of COL11A1 in Chemotherapy Resistance

COL11A1 is one of the top genes in the gene signature that predicts outcome to standard chemotherapy in ovarian and other cancers. A study by Wu et al (2015) shows that ovarian cancer patients who did not respond to standard platinum-based chemotherapies express elevated levels of COL11A1 [65], where COL11A1 was one of the top overexpressed genes. Furthermore, high levels of COL11A1 protein secretion has been linked with resistance to platinum-based therapies in ovarian cancer [69]. More recently, high levels of circulating COL11A1 has been identified in patients with NSCLC [70] and breast cancer [71], which correlated with increased aggressiveness of the disease.

It is interesting to note that the expression of COL11A1 is elevated post chemotherapy in several cancer types and can mediate resistance to cisplatin chemotherapy (Table 1). In lung cancer specimens it has been observed that COL11A1 expression is increased in recurrent tumors [47]. In lung cancer cell lines, elevated COL11A1 expression also mediates resistance of cancer cells to cisplatin, a commonly used chemotherapy drug. Similarly, in ovarian cancer specimens, the expression of COL11A1 is the highest in recurrent tumors compared to primary and metastatic tumors, suggesting that COL11A1 promotes tumor recurrence post chemotherapy [14,24]. We, and others, have shown in human ovarian cancer cell lines and xenograft mouse models that COL11A1 is not only associated with poor response to cisplatin, but also confers cisplatin resistance though multiple mechanisms. We published that COL11A1 upregulates Inhibitors of Apoptosis Proteins (IAP), particularly, XIAP, BIRC2 and BIRC3, expression to evade cisplatin-induced apoptosis [63]. More recently, we identified a novel role of COL11A1 in modulating tumor cell metabolism. COL11A1 signaling in ovarian cancer cell lines makes them more addicted to fatty acid metabolism to drive cisplatin resistance [64]. This ability of COL11A1 to alter the metabolic adaptation of cancer cells benefits the cancer cells to survive the harsh environments generated by chemotherapy. Nevertheless, on the other hand it uncovers new vulnerabilities that offer valuable therapeutic potential. Other groups investigated the mechanism underlying COL11A1-mediated chemoresistance and identified that COL11A1 regulates TWIST1 expression to confer cisplatin and paclitaxel resistance [62]. In another study by Wu et al., c/EBPβ (CCAAT-enhancer-binding protein-beta) has been identified as a transcription factor upregulating COL11A1 expression post chemotherapy [65]. Given that we and others have observed an increase in COL11A1 expression in ovarian and other cancer types, post-chemotherapy, this study is particularly informative because it provides an in-depth understanding of the factors that drive COL11A1 expression in drug-resistant cancer cells that might ultimately cause recurrence.

### 5.2. Role of COL11A1 in Immune Cell Regulation and Immunotherapy Resistance

Given that regulation of immune cell infiltration by ECM dynamics will have profound implications in the response of tumors to immunotherapy and the clinical outcome, it is not surprising that COL11A1 might regulate antitumor immune response. It has been shown that COL11A1-matrisome signature positively correlates with Treg and TH2 signatures and poor clinical outcome in ovarian cancer specimens [45]. However, the enrichment of immune cells in the TME is expected to be diverse and cancer specific. In fact, Wu et al (2020) show that COL11A1 expression in colon adenocarcinoma positively correlates with infiltration of CD4^+^T and CD8^+^T cells, tumor-associated macrophages, neutrophils, and dendritic cells [72]. Whether the presence of these immune cells in the colon TME affects antitumor immunity, has not been investigated in this study. In another study by Lim et al. (2018) [73], the authors report that across 10 tested cancer types, the COL11A1-matrisome signature was associated with a general trend toward abundance of M0 and M1 macrophages, neutrophils, activated mast cells, regulatory T cells (Treg), and T follicular helper (Tfh) cells, activated CD4+ memory T cells, and a decrease of resting CD4+ memory T cells, mast cells, naïve B cells, and resting dendritic cells. In addition, the authors identify a gene signature that predicts response to PD1 immunotherapy. Intriguingly, it has been observed that there is a significant negative correlation between the abundance of “COL11A1-matrisome” (29 gene signature where COL11A1 was one of the top upregulated gene) and response to PD1 checkpoint immunotherapy [43,73]. These predictions, although needing further validation, show a great promise to find biomarkers for immunotherapy response that take into consideration tumor, TME, and host immunity.

## 6. COL11A1 Signaling

### 6.1. Upstream Regulators

There is evidence that cancer cells that are resistant to the commonly used cancer therapeutic drugs, express high levels of COL11A1 [57,65]. Wu et al. [65] demonstrate that cisplatin and paclitaxel can induce COL11A1 expression in the cisplatin-resistant ovarian cancer cell line A2780CP70. Furthermore, the authors of this study also show that knock down of the transcription factor c/EBPβ attenuates the enhancement of COL11A1 expression in A2780CP70 cells treated with cisplatin or paclitaxel and that PDK1/Akt signaling activation upregulates c/EBPβ/COL11A1 expression in these cells. Interestingly, Matsuo et al. [74] not only show that the transcription Factor CCAAT-binding Factor CBF/NF-Y positively regulates the transcription of COL11A1 in A204 cells (a human rhadomyosarcoma cell line) by directly binding to the promoter region, but also show that c/EBP does not bind to the promoter region of COL11A1 in this cell line. This suggests that COL11A1 transcription can be regulated differentially depending on cell type. Members from this group show in a later study that NF-YA also regulates the promoter region of COL11A1 in rat chondrosarcoma (RCS) as well as mouse pre-chondrocyte ATDC5 cells [75], and also show in another study that transcription factor Sp1 also regulates COL11A1 expression in rodent RCS cells [76]. Another transcription factor that is also known to regulate COL11A1 expression in a cancer context is B-myb. Jin et al. [60] show that B-myb is upregulated in NSCLC and B-myb overexpression in H1299 cells leads to COL11A1 overexpression.

In line with studies that demonstrate NF-Y transcription factor positively regulates COL11A1 expression, Wu et al. [33] show that TGF-β1 treatment leads to increased COL11A1 expression through NF-YA in A2780 and OVCAR4 human ovarian cancer cell lines, which is in agreement with another publication that demonstrate that TGF-β1 stimulation enhances COL11A1 expression in fibroblasts as well [77]. In addition to TGF-β1, another extracellular factor that has been shown to upregulate COL11A1 in fibroblasts is the glycoprotein microfibril-associated protein 5 (MFAP5), which has been shown to enhance COL11A1 expression in ovarian fibroblasts [66]. Another factor that regulates COL11A1 expression is Fibroblast Growth Factor-14 (FGF-14). FGF-14 is downregulated in lung adenocarcinoma patient samples [61], and the overexpression of FGF-14 in the lung adenocarcinoma cell line A549 results in downregulation of COL11A1 expression.

Other factors may also influence COL11A1 expression, such as microRNAs. Yang et al. [56] demonstrate that overexpression of microRNA-145 (miR-145) downregulates COL11A1 expression and other chondrocyte markers in the mouse fibroblast cell line C3H10T1/2, while suppression of this microRNA enhances COL11A1 expression. Another microRNA, miR-139-5, has also been shown to suppress COL11A1 expression in the breast cancer cell line ZR-75-1 [78]. There is also evidence that miR-29 downregulates COL11A1 expression during zebrafish development [79]. Although there is no study that has directly showed that miR-29 downregulates COL11A1 in mammalian cells, there are at least two studies that predict that miR-29 downregulates COL11A1 in a mammalian cell context [80,81].

Aside from ovarian/breast cancer cells, sarcoma cells, and fibroblasts, a few other studies have documented how COL11A1 transcription is regulated in osteocytes and myoblasts. In Kahler et al [82], lymphocyte enhancer-binding factor 1 (Lef1)-suppressed MC3T3 pre-osteoblasts show downregulation of COL11A1 expression. The authors of this study demonstrate that administration of Lef1 ligand stimulates COL11A1 transcription in a mouse myoblast cell line C2C12. Finally, Gorski et al. [83] demonstrate that inhibition of a proprotein convertase Subtilisin Kexin Isozyme-1 (SKI-1) leads to decreases in COL11A1 gene expression in UMR106-01 rat osteogenic cell line. This study also demonstrates that SKI-1 activates SREBP-1/2 and CREB H family transcription factors, although they do not directly show that these transcription factors are necessary to induce COL11A1 transcription.

**Figure 2:**
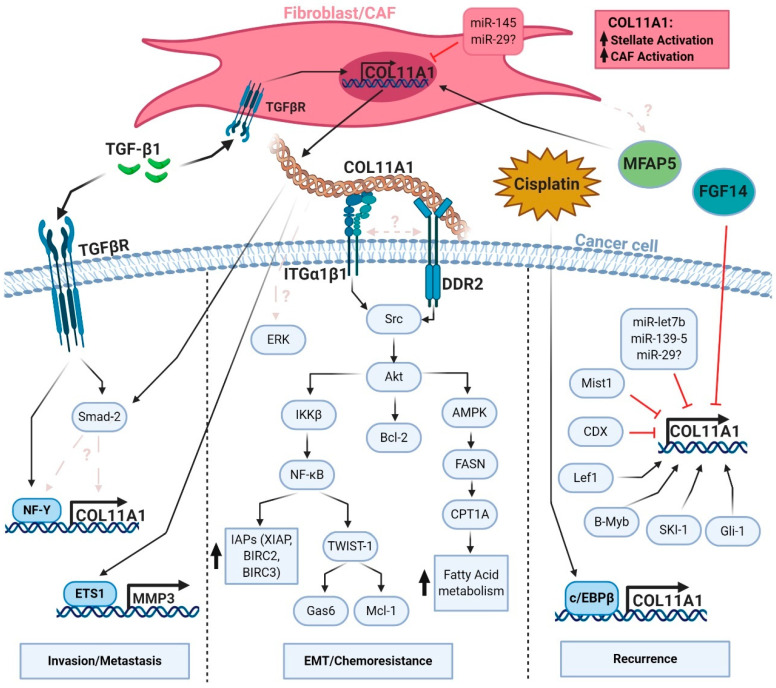
Regulation of COL11A1 signaling in cancer.

In summary, COL11A1 transcription is driven by the transcription factors c/EBPβ, NF-Y, Sp1, and B-myb, and growth factors like TGF-β1 in ovarian cancer, sarcoma cells, and lung cancer cells. Transcription of COL11A1 is also upregulated in fibroblasts, osteocytes, and myoblasts by TGF-β1, MFAP5, Lef1, and SKI-1 treatment. Additionally, microRNAs miR-145, miR-139-5, (and potentially miR-29) as well as the growth factor FGF-14 negatively regulate COL11A1 expression in ovarian fibroblasts, breast cancer cells, and lung cancer cells, respectively (Figure 2).

### 6.2. Receptors

Collagens bind to several cell surface proteins including specific integrin heterodimers (alpha integrin subunits α1, α2, α10, α11 paired with the β1 integrin subunit), the receptor tyrosine kinases Discoidin domain receptors 1 and 2 (DDR1/2), and some other unique cellular receptors including Glycoprotein VI, LAIR-1, and members of the mannose receptor family [84,85,86,87,88]. We and others have shown that ovarian cancer cells bind to COL11A1 through the integrin heterodimer α1β1 and DDR2 [63,64,89]. In Rada et al. [63], we found that ES2 human ovarian cancer cells could not adhere as effectively to COL11A1 when these cells did not express integrin (ITG) subunits α1 and β1, or if these cells did not express DDR1 or DDR2, compared to control ES2 cells. Interestingly, knockdown of ITGα2 did not significantly impair ES2 cell adhesion to COL11A1. In addition to mediating cell adhesion to COL11A1, we and others have shown that COL11A1 promotes ovarian and pancreatic cancer cell survival and chemotherapy resistance through ITGα1β1 and DDR2 in vitro [63,64,89]. Curiously, in our studies, single knockdown of a either integrin α1β1 or DDR2 has been sufficient to attenuate most COL11A1 downstream signaling in ES2 ovarian cancer cells, although DDR2 knockdown caused more inhibition of COL11A1 downstream signaling than ITGα1 knockdown. These results suggest that DDR2 may mediate COL11A1 signaling more dominantly than integrin α1β1 in ovarian cancer cells. However, it is less clear in Wang et al. [89] if single knockdown of ITGα1β1 and DDR2 either fully or partially attenuates COL11A1 signaling across their pancreatic cancer cell lines. There is also the possibility that ITGα1β1 and DDR2 may potentially regulate each other’s expression and/or function, possibly through interacting with each other at the cell’s surface. Xu et al. [90] show that although integrins α1β1 and α2β1 mostly mediate firm adhesion of HEK293 cells to collagen type I, overexpression of DDR1 or DDR2 enhances these cells’ ability to bind to collagen type I despite these receptors not mediating firm adhesion in the absence of integrins α1β1 and α2β1. Furthermore, the authors also show that, for the most part, DDRs do not significantly change surface expression of integrins α1, α2, and β1 compared to control cells (although DDR2-expressing HEK293 cells slightly increase surface expression of integrin α1). However, enhancement of integrin adhesion to collagen type I by DDRs is dependent on DDR activation. 

Currently, no study has shown direct binding of any collagen receptors to specific sequences on the COL11A1 protein. However, Wang et al. [89] propose that integrin α1β1 may bind to the amino acid sequences GFOGER, GROGER, GASGER on COL11A1. This is in line with α1β1 collagen-binding motifs suggested by Xu et al. [90]. GVMGFO is a peptide sequence on type I collagen that DDR2 binds to [90]. However, it appears that COL11A1 lacks this amino acid sequence, suggesting that DDR2 binds to COL11A1 through some other binding motif.

Finally, although no study has tested whether integrin α11β1 can bind to COL11A1, the gene expression of integrin subunit α11 has been shown to correlate strongly with COL11A1 gene expression in a number of different cancer studies [70,91,92]. Of note, integrin α11β1 expression seems to be expressed mostly by fibroblasts and other stromal cells [93], and it is unknown whether cancer cells typically express, or express at all, integrin α11.

In summary, emerging evidence suggests that integrin α1β1 and DDR2 mediate COL11A1 signaling. Further studies are warranted to determine which COL11A1 binding motifs interact with integrin α1β1 and DDR2, if there is any interaction or regulatory signaling between ITGα1β1 and DDR2, and if there are other collagen receptors that can bind to COL11A1 and mediate its signaling (such as DDR1 and integrin α11β1) (Figure 2).

### 6.3. Downstream Effectors

COL11A1 overexpression has been observed in many cancer types [13,14] and has also been shown to stimulate pro-survival signaling across different cancer types as well. We have shown that COL11A1 enhances Src/Akt/NFκB activation in human ovarian cancer cell lines, leading to upregulation of inhibitor of apoptosis protein (IAP) expression (BIRC2, BIRC3, XIAP) [63]. Another group has also found that COL11A1 activates Akt/NFκB signaling in multiple human ovarian cancer cell lines [62,94]. Wu et al. [62] show that COL11A1 activates NFκB signaling by upregulating the expression of IKKβ, a positive regulator of NFκB [95]. The authors of this study also demonstrate that COL11A1 also upregulates expression of TWIST1 (an EMT-promoting transcription factor [96]), and Mcl-1 (a Bcl-2 family pro-survival protein [97]), and GAS6 (a secreted protein promoting cancer cell proliferation [98]), through upregulation of IKKβ and subsequent activation of NFκB in several human ovarian cancer cell lines [62]. Interestingly, this group did not see an induction of the pro-survival proteins BIRC2, BIRC3, nor Bcl-2 when COL11A1 was overexpressed in A2780 and ES2 ovarian cancer cell lines, nor a reduction of these proteins when COL11A1 expression was knocked down in A2780CP70 ovarian cancer cell line.

It has been shown that COL11A1 promotes survival signaling in other cancer types as well. For example, Tu et al. [50] demonstrate that COL11A1 knockdown decreases the colony forming ability of the NSCLC cell line H1299 and also significantly lowers the expression of Bcl-2, CyclinD1, CDK2 and CDK-4 in these cells. The authors also demonstrate that COL11A1 knockdown in these cells decreases the expression of EMT markers vimentin and Snail as well as activities of Akt and ERK. Another group show that COL11A1 enhanced cell proliferation, migration, invasion, and cisplatin resistance of human NSCLC cell lines H520 and H23 [47]. Interestingly, this group proposed that COL11A1 promotes these cellular phenotypes through its activation of the Smad pathway. The authors show that inhibition of the Smad pathway with LND-193189, a Smad signaling inhibitor, attenuates COL11A1-mediated increase of NSCLC proliferation, migration, invasion, and cisplatin resistance in vitro [47]. Wu et al. [33] show that induction of COL11A1 expression is correlated with Smad-2 phosphorylation post TGFβ1 treatment in A2780 and OVCAR4 human ovarian cancer cell lines. However, it is unclear if Smad-2 phosphorylation is required for TGFβ1-induced COL11A1 expression, or if Smad-2 phosphorylation is downstream of the COL11A1 signaling, or possibly both. Zhang et al. [51] show very similar findings to Tu et al. [50] in that knockdown of COL11A1 decreases cell colony forming abilities, migratory capabilities, and Akt and ERK signaling activities in an esophageal squamous cell carcinoma (ESCC) cell line KYSE510. They also note a reduction in the expression of c-MYC and EMT markers vimentin and Slug upon COL11A1 knockdown. Wang et al. [89] show that COL11A1 promotes cell proliferation in pancreatic cell lines (BxPC-3, Capan-1, Mia PACa-2, and PANC-1). In this study, COL11A1 increases BCL-2 expression while decreasing BAX expression through activation of Akt/CREB signaling in BxPC-3 human pancreatic cancer cell line. Interestingly, this group did not see ERK activation by COL11A1 in this cell line, suggesting that COL11A1 may activate different signaling programs depending on the cancer type. Finally, we have discovered that COL11A1 switches the metabolic profile of ovarian cancer cells to fatty acid metabolism. In Nallanthighal et al [64], we demonstrate that COL11A1 upregulates the expression of rate-limiting enzymes of both fatty acid oxidation (CPT1A) and fatty acid synthesis (FASN) in multiple human ovarian cancer cell lines. Upregulation of these fatty acid metabolism enzymes by COL11A1 is mediated by activation of Src, Akt, and AMPK.

In summary, these studies suggest that COL11A1 activates survival signaling in cancer cells, with most studies agreeing that COL11A1 activates Akt to promote cell survival. A couple of studies have shown that COL11A1 activates NFκB signaling, and multiple studies have shown that COL11A1 induces EMT markers and promotes proliferative and migratory phenotypes of cancer cells. However, there is some controversy as to which downstream molecules COL11A1 upregulates/activates. For example, while we and other groups suggest that COL11A1 upregulates IAPs (XIAP, BIRC2, BIRC3) or Bcl-2 [50,89], Wu et al. [62] did not find that COL11A1 upregulates IAPs nor Bcl-2 expression. Additionally, two groups have found that COL11A1 enhances ERK activation [50,51] whereas other group did not observe ERK activation by COL11A1 [89]. Discrepancies between study results could be due to differences in cancer types, cell types, or experimental conditions where COL11A1 levels are manipulated. Further investigation is warranted to define more COL11A1 downstream effector molecules.

It should be noted that most of COL11A1 signaling molecules are also critical regulators of cancer stemness phenotype (e.g., TGFβ, TWIST, c/EBPb, DDR2, Akt, Scr). FAO, a metabolic pathway upregulated by COL11A1, has been shown to be a driver of cancer stemness as well [99]. Therefore, it is very likely that COL11A1 promotes cancer stemness, although there are no studies yet that have confirmed the role of COL11A1 in cancer stemness.

## 7. Targeting COL11A1 Signaling

Although there is no current therapy specifically designed to target COL11A1 in a cancer context, there are certain drugs that target either upstream or downstream molecules of COL11A1, some of which have been or are currently being tested in clinical trials (Table 2). TGF-β, a growth factor known to induce COL11A1 expression, inhibition has been explored as an anti-cancer therapy in the clinic. For example, the TβRI (TGF beta receptor 1) kinase inhibitor galunisertib has been evaluated in two phase II clinical trials for hepatocellular carcinoma (HCC), and treatment with galunisertib showed improvement in overall survival in both clinical phase II trials [100]. Solanum incanum extract (also known as SR-T100), has been shown to downregulate c/EBPβ and COL11A1 expression, thereby sensitizing melanoma and ovarian cancer cells to cisplatin [101]. In addition to targeting COL11A1 transcription, it might also be possible to target COL11A1 post-translation. HSP47 has been shown to be a molecular chaperone required for procollagen folding in the ER [102]. Ito et al. [103] screened small-molecule compounds that inhibit Hsp47’s interaction and chaperone activity with collagen and found that a compound AK778, and its cleavage product Col003, disrupted HSP47/collagen binding and also inhibited collagen secretion. It should be noted that it is unclear whether HSP47 inhibition would also prevent COL11A1 secretion. 

Targeting COL11A1 receptors could be another attempt to inhibit COL11A1’s pro-survival signaling that confers chemoresistance to cancer cells. Dasatinib inhibits DDR2 and Src signaling [104,105], one of COL11A1′s receptors and downstream effectors, respectively. Although dasatinib is well tolerated in patients in phase I/II clinical trials [106], dasatinib treatment alone or in combination with conventional therapies has only shown modest clinical efficacy. In one phase III clinical trial for metastatic castration-resistant prostate cancer (mCRPC), dasatinib in combination with docetaxel showed no significant increase in overall survival compared to doctexal alone, although less bone metastasis has been observed in patients who received combination therapy [107]. Another phase II clinical trial for triple negative breast cancer, which has been terminated early due to slow accrual, showed some modest clinical efficacy for dasatinib [108]. Paclitaxel and dasatinib combination had a 3% complete response rate and a 20% partial response rate out of 32 enrolled patients who continued with the trial. An inhibitor of integrin α1β1 (SAN-300), another collagen receptor that has been shown to mediate COL11A1 signaling, was used in a phase II clinical trial for rheumatoid arthritis (NCT02047604) [109]. However, SAN-300 has not been used for cancer clinical trials as of yet, despite some promising results of other integrin-based cancer therapies.

**Table 2:** Potential inhibitors of COL11A1 signaling.

Drug	Targeted Signaling Molecule	Potential Effects of the Drug in COL11A1 Signaling	Clinical Status	Reference
Galunisertib	TβRI kinase	Downregulates COL11A1 expression	Improved OS from phase II clinical trial for HCC	[100]
SR-T100	c/EBPβ	Downregulates COL11A1 expression	Increased chemosensitization in preclinical studies in melanoma and ovarian cancer	[101]
AK778	HSP47	Inhibits COL11A1 secretion	No cancer trials	[103]
Dasatinib	DDR2/Src kinase	Inhibits COL11A1 binding and signaling activation	Well tolerated (phase I); modest clinical efficacy (phase II); less bone metastasis but no OS improvement (phase III) for different cancers	[106,107,108]
SAN-300	α1β1 integrin	Inhibits COL11A1 binding and signaling activation	No cancer trials; well tolerated in phase II clinical trial for rheumatoid arthritis	[109]
AZD5363	Akt	Inhibits COL11A1-induced chemoresistance	Improved PFS in clinical trials for multiple cancers	[110]
SC66	Akt	Inhibits COL11A1-induced chemoresistance	Increased chemosensitization in xenograft mouse models	[94]
ASTX660	IAP	Inhibits COL11A1-induced chemoresistance	Well tolerated in phase I cancer trials	[111]
APG-1387	IAP	Inhibits COL11A1-induced chemoresistance	Well tolerated in phase I clinical trials for solid tumors	[112]
LCL161	IAP	Inhibits COL11A1-induced chemoresistance	Well tolerated in phase II trial in myelofibrosis (a form of leukemia)	[113]
Birinapant	XIAP/cIAP1	Inhibits COL11A1-induced chemoresistance	No clinical benefit in phase II trial for high grade serous ovarian cancer	[114]
Etomoxir	CPT1 (FAO enzyme)	Inhibits COL11A1-induced chemoresistance	No cancer trials; phase II trial for diabetes and heart failure suspended due to liver and renal toxicity	[115]
Perhexiline	CPT1 (FAO enzyme)	Inhibits COL11A1-induced chemoresistance	No cancer trials; FDA approved for cardiac diseases	[116]
Ranolazine	ACAA2 (FAO enzyme)	Inhibits COL11A1-induced chemoresistance	No cancer trials; FDA and EMA approved for angina	[116]
Trimetazidine	ACAA2 (FAO enzyme)	Inhibits COL11A1-induced chemoresistance	No cancer trials; EMA approved for angina	[116]

Aside from targeting upstream regulators and receptors of COL11A1, there have been clinical trials that have targeted other COL11A1 downstream effector molecules. Akt inhibition has been shown to attenuate COL11A1 signaling and cisplatin resistance in ovarian cancer cells [63,64,65,94], making it an obvious COL11A1 downstream effector molecule to target. However, clinical trials using Akt inhibitors to treat a multitude of cancers has given both promising and disappointing results, as well as some undesired side effects including severe hyperglycemia [117]. One promising Akt inhibitor is AZD5363, which was shown to be effective against solid tumors harboring Akt1 E17K mutation [110]. It is also noteworthy that Wu et al. [94] has demonstrated that the Akt inhibitor SC66 attenuates COL11A1-driven chemoresistance in human ovarian cancer cells in vitro and in vivo, making SC66 a promising drug candidate to treat COL11A1-high cancers. Targeting inhibitor of apoptosis (IAPs) proteins, which have been shown to be upregulated by COL11A1 [63], might be another way to target the COL11A1 signaling. Two phase I clinical trials have demonstrated that IAP inhibitors ASTX660 and APG-1387 were well tolerated by patients [111,112]. Another IAP inhibitor LCL161 (A Smac mimetic) was also well tolerated in a phase II clinical trial for myelofibrosis, a form of leukemia [113]. Another clinical trial using birinapant, an XIAP/cIAP1 antagonist, to treat high-grade serous ovarian cancer showed that birinapant was well tolerated and downregulated tumor cIAP1 [114]. However, accrual of patients into the study was stopped due to insignificant clinical benefit of treatment.

Finally, targeting fatty acid metabolism may be another way to target COL11A1 signaling in a clinical context. Although inhibition of COL11A1-induced FAO sensitized ovarian cancer cells to cisplatin treatment in vitro [64], there have been no clinical trials that use FAO inhibitors for cancer treatment yet. Clinical trials using the CPT1 inhibitor etomoxir for diabetes and heart failure was retired due to liver and heart toxicities [115]. More promising FAO inhibitors including perhexiline, ranolazine, and trimetazidine, which are currently being used to treat angina pectoris [116], could potentially be repurposed to treat COL11A1-high cancers.

In summary, although there is no therapy designed to directly target COL11A1, potential therapeutic strategies for COL11A1-high cancers include targeting COL11A1 transcription by inhibiting TGF-β and c/EBPβ (Galunisertib/SR-T100), COL11A1 secretion (HSP47 inhibitor AK778/Col003), COL11A1 receptors DDR2 and integrin α1β1 (Dasatinib and SAN-300), Akt activation (AZD5363, SC66), IAPs (Birinapant), or FAO enzymes (Perhexiline, ranolazine, and Trimetazidine). It should be noted that although some of the listed drugs initially showed limited clinical efficacy, it is possible to improve their outcome considering the following parameters. Firstly, it is expected that patients whose tumors show high COL11A1 expression might be suitable candidates for treatment with these drugs. Secondly, devising multi-drug combinations that target several of the COL11A1 signaling molecules simultaneously are more likely to achieve a successful clinical response. Furthermore, there is a need for identification of more specific COL11A1 effector molecules to avoid systemic toxicity and increase specificity.

## 8. Remaining Challenges

Despite significant advancement of our knowledge on the biological functions of COL11A1 in cancer progression and the underlying mechanisms, there are still many questions to be addressed. For example, COL11A1 overexpression has been associated with poor prognosis majorly in solid tumors such as breast, ovary, lung, stomach cancers [13]. However, COL11A1 overexpression in hematological malignancies including AML, CLL, B-ALL, and DLBCL is correlated with better prognosis [14]. Given the lack of information on the role of COL11A1 in hematological malignancies, it is unknown at this point why COL11A1 functions differently in solid vs. hematological cancers. With regard to the structure of COL11A1, it is unknown which two alpha chains assemble with COL11A1 in a cancer setting. Also, the proteins bound to the N-terminal and C-terminal domains of COL11A1 in different cancer types are unknown. In addition, it is not clear whether the COL11A1, secreted by cancer cells and CAFs, is structurally and functionally similar. More evidence is needed to show how COL11A1 regulates biophysical properties of tumor ECM and how it affects migration of tumor and immune cells. Similarly, biological functions of COL11A1 in other stromal cell types are largely unknown especially in vivo due to a lack of genetically engineered mouse models. Despite our increased understanding of COL11A1 functions in chemoresistance, the roles of COL11A1 in cancer stemness, tumor dormancy, inflammation and recurrence are still unclear. For example, although studies in pancreatic cancer have highlighted the role of COL11A1 as a cancer-specific but not inflammation-specific biomarker, COL11A1 overexpression has been identified in chronic inflammatory diseases like osteoarthritis [118]. Furthermore, COL11A1 signaling has been shown to upregulate NFκB, a known proinflammatory transcription factor. These findings support the hypothesis that COL11A1 might regulate inflammation in a cell type-specific, tissue-specific and/or a cancer stage-specific manner. Given the complexity of interactions between cells of the TME, there are significant knowledge gaps in understanding how COL11A1 signaling cross-talks with other signaling pathways and whether this crosstalk can modulate cancer phenotype. Lastly, there is very little information about the 28 different collagen subtypes whose functions in cancer might be unique or redundant. This could limit the therapeutic efficacy of drugs if other collagens could compensate for the loss of COL11A1. Therefore, it is critical to address what unique functions and mechanisms are engaged by COL11A1, compared to other collagen subtypes.

## 9. Conclusions

In summary, the expression of COL11A1 is elevated in several cancers and is associated with poor survival, chemoresistance, and recurrence. COL11A1 expression has been detected in not only tumor cells, but also other tumor-associated stromal cells. Through binding to specific receptors and activating several key cell-survival signaling pathways, COL11A1 can promote cancer progression, metastasis and drug resistance. COL11A1 can also be used as a biomarker to identify patients who have a high risk of developing chemoresistance and tumor recurrence. Patients expressing high levels of COL11A1 could be treated by pharmaceutical inhibitors blocking COL11A1 signaling to attenuate tumor cell chemoresistance and recurrence. Further study of COL11A1 will facilitate the design of personalized targeted therapies to improve clinical outcome in many cancer types.

## Figures and Tables

**Table 1:** COL11A1 expression and functions in different cancers.

Type of Cancer	Role of COL11A1	Mechanism	References
Breast	increases drug resistance; upregulates EMT; increases cancer cell invasion and metastasis in vitro and tumor growth in vivo	suppresses CDX2/microRNA let-7b expression	[55]
		miR-139-5 suppresses COL11A1 expression	[56]
		Binds to OSM to activate STAT signaling	[23]
Colorectal	one of the genes of the “invasion gene signature”; associated with poor survival	TGFβ is an upstream regulator of COL11A1	[29]
		co-expressed with COL5A2	[26]
Esophageal	increases cancer cell proliferation, migration, and invasion in vitro; positively correlates with advanced clinical stage, invasion depth and lymph node metastases	upregulates EMT in an AKT/ERK/c-Myc dependent manner	[51]
Gastric	offers poor prognosis; increases cancer cell proliferation, migration and invasion	upregulates several proliferation genes including CDK6, TIAM1, ITGB8 and WNT5A	[57]
Glioma	offers poor prognosis; increases neural stem cell migration to tumor sites and metastasis	activates ERK-dependent migration	[58]
		hyperacetylates histone H3 and upregulates transcription of tumorigenic factors	[59]
Head and neck	offers poor prognosis; upregulates cancer cell proliferation, migration, and invasion		[49]
Lung	offers poor prognosis; increases cancer cell proliferation, migration, invasion and drug resistance	upregulates Smad 2 signaling	[47]
		upregulates p-AKT, p-PI3K and p-ERK	[50]
		B-myb is a transcription factor that increases COL11A1 expression	[60]
		FGF14 upregulates COL11A1 expression	[61]
Ovary	offers poor prognosis; upregulates EMT; increases cancer cell invasion, metastasis, and drug resistance in vitro; increases tumor growth in vivo	TGFβ is an upstream growth factor that induces COL11A1 expression	[14,24,33]
		remodels ECM via Ets-1/MMP3 signaling	[62]
		mediates tumor-stroma interaction via α1β1 integrin and DDR2 receptors	[63,64]
		upregulates Akt1/c/EBPβ signaling	[65]
		upregulates IkkB/NFκB-TWIST1 axis	[62]
		upregulates IAPs in a Src-Akt-NFKB dependent manner	[63]
		upregulates fatty acid metabolism in a Scr-Akt-AMPK dependent manner	[64]
		MFAP5 is an upstream growth factor that increases COL11A1 expression in ovarian CAFs	[66]
Pancreas	associated with poor survival; increases tumor cell invasion, metastasis, and drug resistance in vitro; increases tumor growth in vivo	Mist-1 is a transcriptional repressor of COL11A1	[67]
		Gli1 is a transcriptional activator of COL11A1	[68]
		NF-YA is a transcriptional activator of COL11A1	[30]

## Data Availability

Not applicable.
